# Optimization of Printing Process Variables and the Effect of Post-Heat Treatments on the Mechanical Properties of Extruded Polylactic Acid–Aluminum Composites

**DOI:** 10.3390/polym15244698

**Published:** 2023-12-13

**Authors:** Sakthi Balan Ganapathy, Aravind Raj Sakthivel, Jayakrishna Kandasamy, Tabrej Khan, Mansour Aloufi

**Affiliations:** 1 Department of Manufacturing Engineering, School of Mechanical Engineering (SMEC), Vellore Institute of Technology, Vellore 632014, Tamil Nadu, India; sakthibala33@gmail.com (S.B.G.); mail2jaikrish@gmail.com (J.K.); 2 Engineering Management Department, College of Engineering, Prince Sultan University, Riyadh P.O. Box 66833, Saudi Arabia; maloufi@psu.edu.sa

**Keywords:** additive, annealing, ductility, mechanical properties, heat deflection temperature, post-heat-treatment behavior

## Abstract

Polymer extrusions are employed in the fabrication of crucial parts for automotive, aerospace, and other mechanical applications. The use of fillers and microfibers is contributing to the advancement of material extrusion polymers. In order to enhance their mechanical characteristics, printed materials undergo a post-heating process utilizing microwaves. Specimens were fabricated using polylactic acid filaments containing 2 wt% aluminum. Two sets of specimens were fabricated and subjected to testing in order to evaluate the features of extruded specimens and specimens that underwent post-heating. In terms of mechanical performance, specimens subjected to post-heating exhibited superior results compared to specimens merely subjected to extrusion. The tensile, flexural, and Shore D hardness properties of the specimens exhibited improvements of 5.07, 6.16, and 1.32%, respectively, after being subjected to heating. Furthermore, the wear rate decreased by 13.58 percent. The results of the regression analysis indicate that the print angle and the air gap exhibit the greatest influence on the observed responses. The validation test outcomes exhibit a high level of concordance with the predicted findings. The mechanical and surface properties of components extruded with filler-added material are enhanced by subsequent heating.

## 1. Introduction

The domain of three-dimensional printing, often known as additive manufacturing, is now experiencing significant growth and has attracted considerable attention in academic research due to its numerous uses within various industries. One of the key areas of study pertains to the advancement of materials and techniques for diverse applications, encompassing the enhancement of mechanical characteristics and the reduction of weight. Various additive manufacturing processes are increasingly being utilized to enhance or, in certain cases, substitute conventional manufacturing processes. This trend can be attributed to several aspects, such as efficiency in terms of time, cost-effectiveness, and improved accessibility [1]. Fused deposition modelling (FDM) is widely utilized in academia, industry, and individuals. The FDM method is founded on the principle of heating and extruding thermoplastic filament to construct products layer by layer [2,3]. The method of melt mixing, often referred to as melt blending, is frequently used to generate composite filaments for FDM printers. The filament is heated above its melting temperature during the melt mixing process, and additives are then added as the filament is being pushed out [4,5]. These filaments have demonstrated the ability to generate products with improved mechanical properties, and they can also be utilized to implement novel applications like electromagnetic shielding and solvent sensing [6,7]. Various particulates and fibers have been combined with polylactic acid, nylon, or polypropylene as substrates to produce these filaments. In subsequent sections, a more in-depth examination of these filaments is provided. One of the greatest benefits of the FDM process is the ability to adjust numerous parameters that influence the final product’s characteristics. This includes temperature, layer thickness, angle, and fill pattern [8,9]. Optimizing these parameters is essential for attaining the desired mechanical, thermal, or other properties [10,11]. At the same time, the production of customized filaments poses a number of difficulties and print quality issues. To reduce the number of flaws in the printed samples, the printing process parameters must be optimized for each variety of printing material. After printing the specimens, the materials are post-processed to enhance the interfacial adhesion between the printed layers and any blow holes caused by filament overheating. When microwaved at 185 °C for 60 s, 3D-printed PLA/silicon carbide composites’ interfacial bonding is enhanced due to re-melting [12]. Microwave-assisted heating is still used to strengthen a PLA-copper composite with enhanced fracture durability. Specimen exposure to microwaves with 3 kW of power for 75 s has been observed to result in a 37% increase in maximal tensile fracture. The post-heating enhanced polymer crystallinity decreased micro voids and residual stresses and increased the composites’ fracture toughness [13]. Inclusion of each filler will induce different changes in the base materials. The filler type, filler shape, size, filler addition method, manufacturing method, pre- and post-processing methods, and applications are some of the factors that determine the properties of the base materials after filler addition. Some fillers will enhance the strength and some may decrease the strength. The compatibility of the fillers with the base material plays a major role in determining the overall strength of the composite material. The merits of using aluminum as a filler are that it increases stiffness and strength. Additionally, aluminum-filled PLA composites often exhibit improved thermal conductivity, making them suitable for applications wherein heat dissipation is crucial. The presence of aluminum fillers can also enhance the dimensional stability of PLA, making it less prone to deformation. This study demonstrated that differences in infill density and layer height had a substantial impact on the mechanical properties of acrylonitrile styrene acrylate components produced by material extrusion. These attributes include tensile strength, flexural strength, and impact strength [14]. Ceramic and metal fillers and additives can be incorporated into both conventionally and additively manufactured polymer composites in order to enhance their thermal stability and mechanical properties [15]. The printing layer heights and raster angle had the greatest impact on the surface irregularity and delamination factor of PLA-Al composites produced by the FDM method [16]. Very few researchers have recently worked on composites with Al and polylactic acid that are intended for a variety of applications. A recent study has reported on the analysis of the mechanical properties of PLA with the incorporation of 5% aluminum. The study revealed that increasing the printing parameters, such as nozzle temperature and infill density, resulted in improved mechanical properties. According to the process parameters, the failure modes range from ductile to brittle. This work demonstrated the successful reinforcement of metal infills into polymer composites, which can be utilized for many industrial applications [17]. The study involved a method of manufacturing aluminum layers reinforced with PLA to enhance thermal conductivity in the friction stir welding (FSW) process. The PLA-Al composite with alternating layers was manufactured using the fused filament fabrication process, followed by the FSW process. The deposition of Al films between PLA layers was observed to enhance both the crystallinity and mechanical characteristics [18]. A novel methodology was devised, involving the deposition of alternating layers of sprayed aluminum onto PLA. It was shown that the tensile properties were influenced by the number of stacked metal spray layers and the infill density [19]. An investigation was conducted to examine the impact of laser scanning speed on the surface and mechanical characteristics of PLA-Al composites. The laser polishing process was discovered to reduce porosity and enhance the interfacial adhesion between Al fibers and PLA matrix [20]. No studies about the impact of post-heat treatment on the mechanical properties of PLA-Al composites, specifically the hardness and wear resistance of the materials, have been documented in the above literature. Furthermore, there is no previous study that has examined how printing characteristics such as air gap, print angle, and filling pattern affect PLA-Al composites. These aspects render this study innovative and distinctive, offering valuable insights to researchers in the field of metal-filled polymer composites manufactured by additive processes.

The filament utilized in this study is a combination of poly-lactic acid and aluminum. This material’s composition was determined using a scanning electron microscope. This material was printed via the material extrusion process. Variations were made to the print angle, air gap, and fill pattern during the printing process. The tensile, flexural, hardness, and wear rate of nine specimens printed according to Taguchi’s experimental design were calculated. Taguchi’s experimental design was used to determine which variables have the greatest impact on the performance of the specimens.

## 2. Materials and Methods

### 2.1. Materials

**Figure 1:**
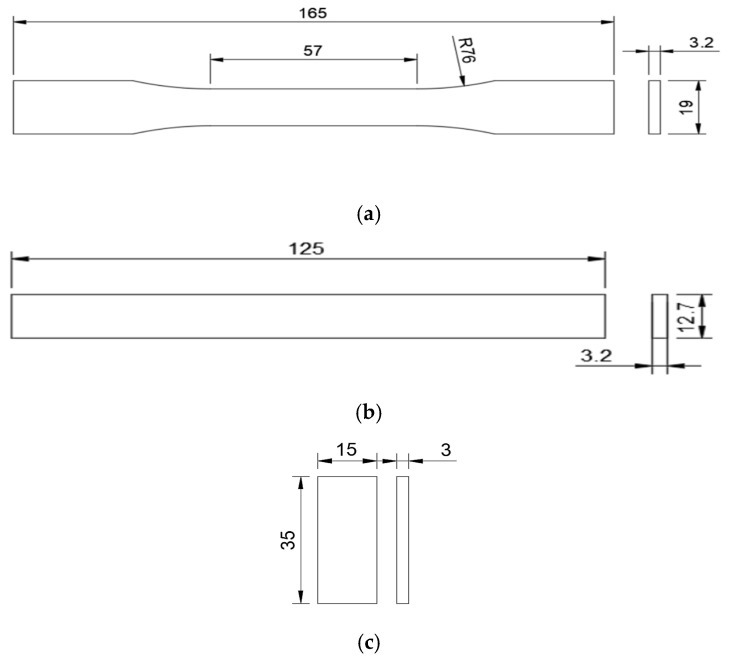
(**a**) Tensile test specimen; (**b**) flexural test specimen; (**c**) hardness test specimen (All dimensions are in mm).

Aluminum was blended with PLA through material extrusion. Metro Composites, Chennai, Tamil Nadu, India, provided the filament for this study, which was produced by Macfos Limited, Pune, India. To produce the filaments, a 2 wt% concentration of aluminum was included in the PLA pellets. Subsequently, the extrusion process was employed to manufacture filaments with a standardized diameter of 1.75 mm. To enhance various qualities of the generated product, additives are incorporated into standard filaments like PLA, and nylon, used in additive manufacturing. PLA was selected for this investigation primarily due to its mechanical properties, melt temperature, and biodegradability [11]. The authors developed this material with the purpose of imparting and examining the electrical and thermal conductivity characteristics in a biodegradable thermoplastic known as polylactic acid. As a first step, the authors looked into the way adding aluminum affects the material’s mechanical properties. Additionally, small percentages were chosen in order to investigate the fundamental changes in the characteristics of the base material. This was also inexpensive and had a relatively low printing temperature, allowing production to be achieved with desktop printers. After printing the specimens with additive included filaments, per the standard dimensions, composite specimens were tested for characteristics. Figure 1a–c indicate the specimen dimensions per ASTM standards for tensile, flexural, and hardness tests, respectively.

### 2.2. Fabrication Method

One of the most popular rapid prototyping technologies is material extrusion, and due to its lower cost, it is used by hobbyists and researchers equally, although for different purposes. For this study, the WANHAO Duplicator 4S, Hangzhou, Zhejiang, China, was used to carry out the material extrusion process. As per the machine specifications, it has a build volume of 225 × 145 × 150 mm, with a maximum extruder temperature of 260 °C, an X, Y axis positioning resolution of 0.02 mm, an X, Y axis max speed of 5000 mm/min, and a Z axis max speed of 1000 mm/min.

The specimens were initially created with computer-aided design software (SolidWorks 2020) in adherence with the criteria set by the American Society for Testing and Materials (ASTM), and saved in the Standard Tessellation Language (STL) format. The STL file was then imported into the slicing software (PrusaSlicer 2.7.0), and the codes were generated after slicing. Using a statistical tool, the experimental input parameters and trial details were established. As per the L9 orthogonal array Design of Experiments (DOE), nine experiments were obtained. Under optimal conditions, the specimens were printed using the input parameters obtained through DOE, and the printed samples were promptly preserved and stored. The samples were printed using a WANHAO Duplicator-model 3D printer. For each test, eighteen specimens were produced, among which nine samples were tested before heat treatment and nine specimens were tested after heat treatment. In total, 72 specimens were produced for all the four tests performed. Printers equipped with heated chambers have a restricted heating capability, which enables them to sustain a consistent temperature throughout the printing process. The objective of the author is to investigate the behavior and transformations that take place in a material when it is exposed to elevated temperatures following the printing procedure. Consequently, the authors opted for this particular printer model for their research, and subsequently, the specimens were heated.

### 2.3. Selected Process Parameters and Their Levels

**Figure 2:**
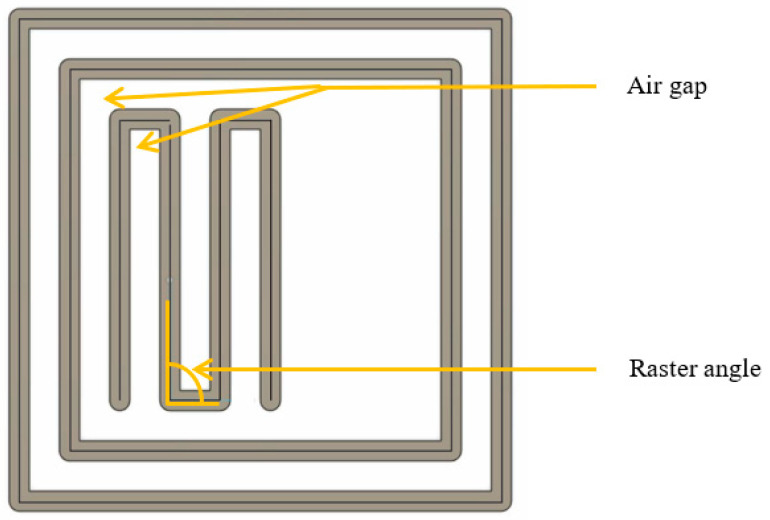
Details about air gap and raster angle.

The printing process parameters have a significant effect on the final product’s characteristics. Numerous studies have examined the variation in one or more mechanical properties as a result of changes in print orientation, infill pattern and percentage, layer thickness, and more [21]. In this investigation, we chose to vary three primary parameters: print angle, air gap, and fill pattern. Three levels of each parameter were used to print specimens in accordance with ASTM specifications for each test. This study optimizes these characteristics for high tensile, hardness, and flexural strength, and wear resistance. In a recent study, raster angle significantly affected specimen ultimate tensile strength [22,23]. This study uses 15, 30, and 45-degree raster angles. Positive, zero, and negative are the three distinct categories. A positive air gap indicates that there is space between two adjacent filaments. A negative air gap is when portions of the two filaments overlap. Zero air gap is when they are in direct contact. A distance of 0.05 mm was provided to maintain the positive and negative air gaps. PrusaSlicer was used to slice the design layer by layer, and for generating the codes. The air gap and raster angle are indicated in Figure 2.

**Figure 3:**
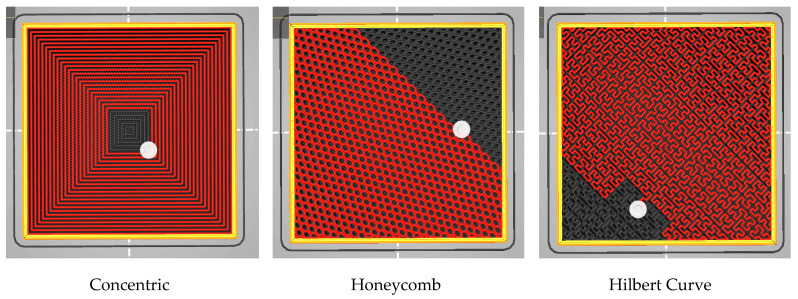
Different types of filling patterns obtained with PrusaSlicer. Red—Printed portion; Grey—Unprinted portion; White—Extrusion nozzle head; Black—Layer gaps.

The method by which the filament is applied to fill the specimen’s interior volume is referred to as the fill pattern. The variability in this study will manifest in the various pathways used for the deposition of materials. Common infill designs include line, rectilinear, grid, triangular, star, cubic, honeycomb, concentric, and Hilbert curve. The infill patterns for this investigation are concentric, honeycomb, and Hilbert curve. Figure 3 was obtained using PrusaSlicer software (PrusaSlicer 2.7.0), and shows the various fill patterns used for this investigation.

Numerous studies have examined how filling patterns and infill density affect specimen mechanical characteristics. Impact strength, elongation, and tensile strength have been discovered to be influenced by the infill density and raster direction [24,25,26]. It has been discovered that the internal architecture or infill pattern affects flexural features, friction coefficient, Poisson’s ratio, and stress values [27,28,29]. The other printing parameters like layer thickness, nozzle temperature, platform temperature and infill density were fixed to 0.10 mm, 220 °C, 50 °C, and 100%, respectively. This study examines how these parameters affect a specimen’s flexural properties, wear resistance, tensile strength, and hardness. The DOE was formed using the parameters and levels indicated in Table 1.

### 2.4. Post Heating and Mechanical Properties’ Evaluation Methods

Taguchi’s experimental design determined how the process parameters affected the printed specimens’ tensile, flexural, wear, and hardness properties. In 1986, Dr. Genichii Taguchi introduced the system of orthogonal arrays. There are numerous orthogonal array designs, but L9 was chosen for this investigation. As the Taguchi method is a fractional factorial design strategy in which only a subset of all possible outcomes are considered, it reduces costs and improves quality. Multiple factors can be adapted simultaneously, and fewer trials can yield more quantitative data [30].

**Figure 4:**
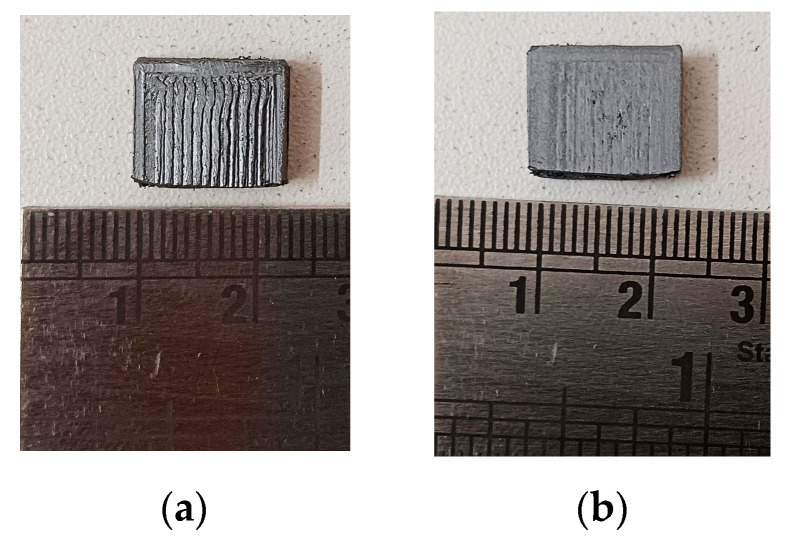
Wear test specimens, (**a**) Before heat treatment, and (**b**) After heat treatment.

It has been utilized in numerous applications, including the development of a method for optimizing friction the parameters of the stir-welding process [31], and the cost and time estimation of projects depending on various parameters [32]. Prior to assessing the mechanical characteristics, a specific set of specimens was selected for a subsequent heating procedure, out of two available sets of specimens. Following the printing process, a single set of nine specimens was assessed in order to determine their inherent characteristics. The subsequent heat treatment was performed using a microwave apparatus, maintaining a consistent temperature of 185 °C for a duration of 60 s. Following a 60 s heating period, the specimens were subsequently cooled to room temperature and subsequently assessed for their mechanical properties. Upon undergoing deformation, polylactic acid (PLA) exhibits the ability to restore its initial shape when exposed to temperatures surpassing its glass transition temperature. The mechanical characteristics and recovery of PLA-printed items can be influenced by temperature and chemical treatments [33]. After using a typical 3D printer to print carbon-fiber-impregnated PLA, the post-heat treatments were carried out via three distinct techniques, including remelting the material in salt, heating it in an oven, and using a microwave oven. The findings demonstrated an increase in rigidity in samples that had been remelted in salt, as well as improvements in bending strength attributes [34]. Figure 4 presents the wear test specimens, with Figure 4a displaying the specimen prior to heat treatment and Figure 4b showcasing the specimen subsequent to heat treatment. A noticeable distinction was observed in the surface characteristics of the specimen. In the non-heat treated specimen, the presence of layer gaps and a stair case effect was evident. Conversely, the heat-treated specimen exhibited a smooth surface with minimum layer gaps, indicating that the layers had fused together.

The capacity of a material to withstand forces that are applied in a tensional state is referred to as its tensile characteristics [35]. The breaking strength of a specimen under the application of force is referred to as the tensile strength of the specimen [36]. The evaluation of tensile strength is of the utmost significance and finds use in a variety of contexts, including the peel testing of air frames, the quality control of fittings, and the determination of the bond strength of adhesives. Flexural strength is a material’s resistance to perpendicular bending forces [37]. In order to determine whether or not a material is suitable for a given application and how long it will last, it is vital to consider this factor. Hardness is a term that describes the resistance of a substance to being indented or deformed in any way [38]. It is primarily utilized for quality control applications, material characterization, and material determination. Wear resistance is a material’s ability to resist mechanical wear. It determines the lifespan of friction-exposed materials [39].

**Figure 5:**
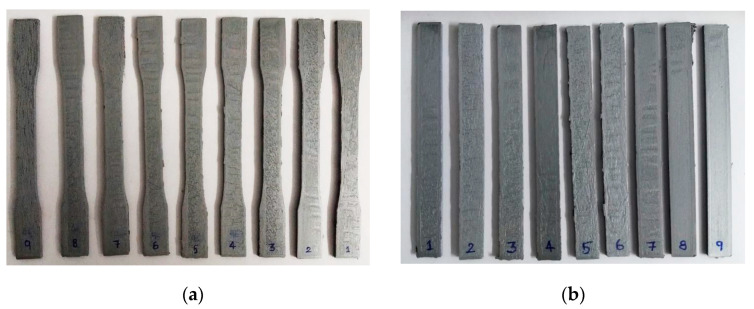
(**a**) Printed PLA-Al tensile specimens; (**b**) printed PLA-Al flexural test specimens.

The testing design and methodology adhered to ASTM standards. ASTM D638 [40] Type I was used to print tensile specimens. ASTM D638 was the most prevalent standard for determining the tensile properties of reinforced and unreinforced plastics [41,42]. Recent years have witnessed a rise in the significance of this standard, which comprises a variety of subtypes with varying specimen dimensions. The flexural test conforms to ASTM D790 standards [43]. The standards are utilized to designate the dimensions of specimens. The standard specifies 3-point flexure tests for rigid, semi-rigid, and fiber composite plastics [44,45]. The test is conducted on a universal testing system at a rate proportional to specimen depth. ASTM D2240 [46] was used to measure sample hardness. The test primarily applies to soft materials, such as plastics and rubbers, and assesses the penetration of a specified indenter under specified conditions [47,48]. The ASTM G99 [49] criteria were utilized in order to evaluate the material’s resistance to wear. Pin-on-disk equipment is used in this method. It has been put to use in the past to conduct tests on a variety of 3D printing materials [50]. Utilizing the Instron 8801 Universal Testing Machine (UTM), Norwood, USA, the tensile and flexural tests were conducted. The UTM has a 100 kN capacity and a 150 mm stroke length. The load was imparted at 2 mm/min cross head speed. The specimens’ hardness was determined using a Shore D hardness analyzer, made by Mitutoyo Corporation, Kawasaki, Japan. The scale ranges from 1 to 100, with 1 indicating lesser hardness and 100 indicating higher hardness. There will be no need for additional calculations as it will provide the results in numerical form. The hardness of each specimen was measured in three distinct locations, and the averages were tabulated. To assess the wear rate of the composite specimen, a pink-on-disk wear-testing apparatus was employed, featuring a European Norm (EN31 [51]) disk and a steel pin. The wear test apparatus utilized in this study was produced by Ducom, a company based in Bohemia, NY, USA. The composite sample was printed as a small segment and affixed to the base of the pin. The mass of the specimen was initially assessed using a scale and subsequently documented. The wear test was conducted with specific operating settings that were predetermined. These parameters included a track diameter of 0.06 m and a disk speed of 1000 rpm, which were maintained for a duration of 15 min. The angular velocity can be calculated using Equation (1). To ensure precise results, a consistent load of 10 N was uniformly imposed on every specimen. Additionally, the disk underwent a thorough cleansing procedure using acetone and ethanol after each test. Equation (2) can be employed to ascertain the sliding distance, whilst Equation (3) can be utilized to compute the volume loss. Following this, the rate of wear can be determined using Equation (4). The rate of deterioration was assessed for both the original and post-heated specimens. In Figure 5, the extruded specimens for tensile and flexural tests are shown.

(1) V=2πRN/60

where

*V* = Sliding velocity (m/s);
*R* = Radius of the wear track (m);
*N* = Rotating speed of the disk (rpm).

(2) S=V×t

where

*S* = Sliding distance (m);
*t* = Sliding time (s).

(3) VL=ML×1000/ρ

where

*VL* = Volume loss (mm^3^);
*ML* = Mass loss (grams);
*ρ* = Density of the material (g/cm^3^).

(4) WR=VL/S

where

*WR* = Wear rate (mm^3^/m).

## 3. Results and Discussion

**Figure 6:**
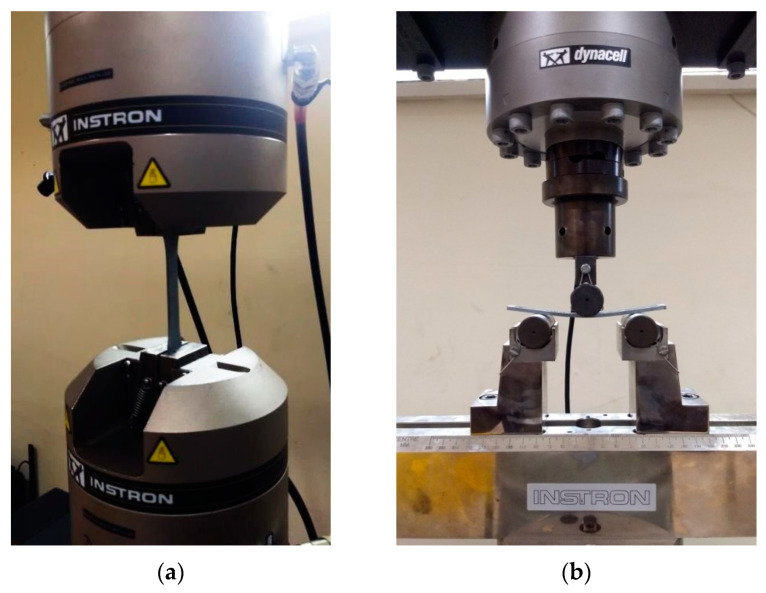
(**a**) Testing of PLA-Al tensile specimens in UTM; (**b**) three-point flexural test of PLA-Al specimens.

In accordance with the experimental design, a total of 18 specimens were produced for each test. Out of these, nine specimens were subjected to direct testing without any heating, while the remaining nine specimens were tested after undergoing a heating process. The identical methodology was employed for conducting tensile, flexural, hardness, and wear tests. The experimental results of the specimens that were examined right after printing and after heating are presented in Table 2 and Table 3, respectively. Figure 6a,b show the test setup that was used for tensile and flexural testing, respectively. It is clear from looking at Table 2 that the ninth specimen, which had been printed with a 45-degree print angle, a negative air gap, and a honeycomb-type fill pattern, possessed the highest Shore D hardness, tensile strength, and flexural strength, while also having the lowest wear rate. A Shore D hardness of 85.7, maximum tensile strength of 44.47 MPa, flexural strength of 80.67 MPa, and a minimum wear rate of 1.32 × 10^−2^ mm^3^/m are the experimental results of the original extruded composite material without post-heating. The experimental results before and after heat treatments are provided in Table 2 and Table 3, respectively.

Besides process parameters, it was clear from Table 3 that the mechanical properties were improved after manufacturing, by means of a heat treatment. Following post-heat treatment, the very same ninth specimen demonstrated a maximum tensile strength of 46.77 MPa, a flexural strength of 86.21 MPa, a Shore D hardness of 86.86, and a minimum wear rate of 1.09 × 10^−2^ mm^3^/m. The tensile and flexural strength of the pure PLA without any additives were found to be 60 MPa and 59.6 MPa [21,52]. When compared to pure PLA results, the PLA-Al results were found to be better in flexural properties, and the tensile strength was found to be reduced.

**Figure 7:**
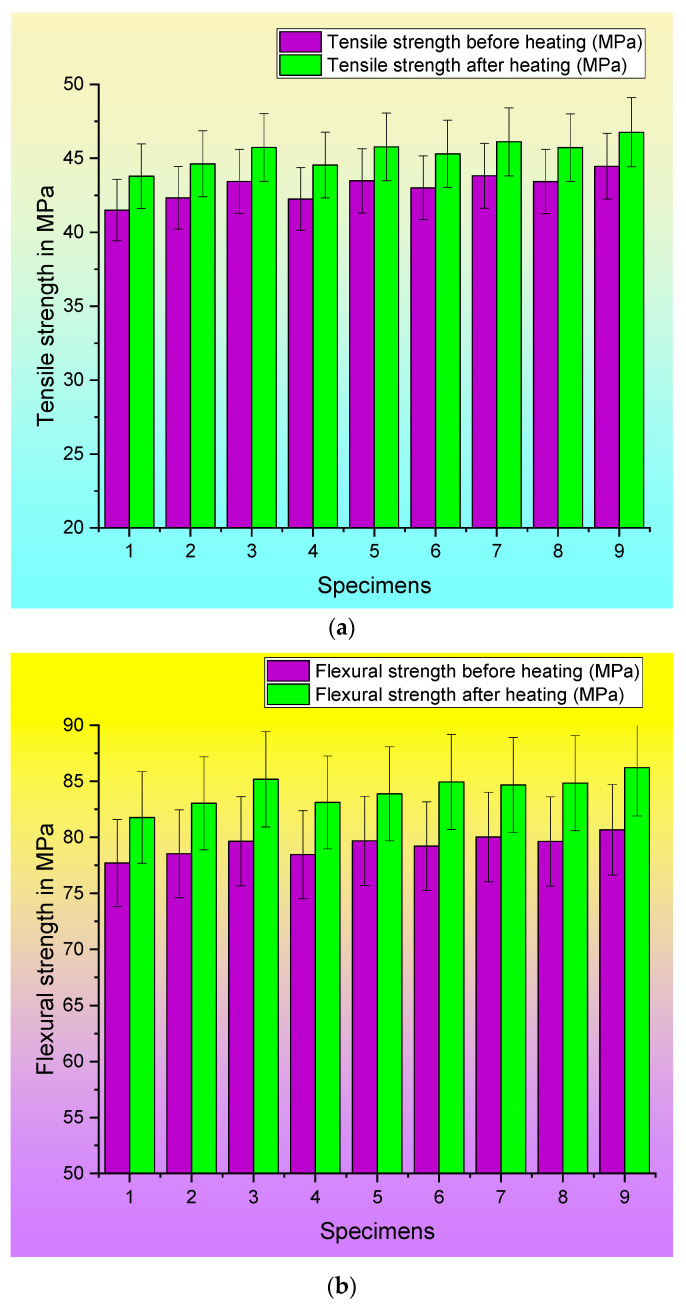

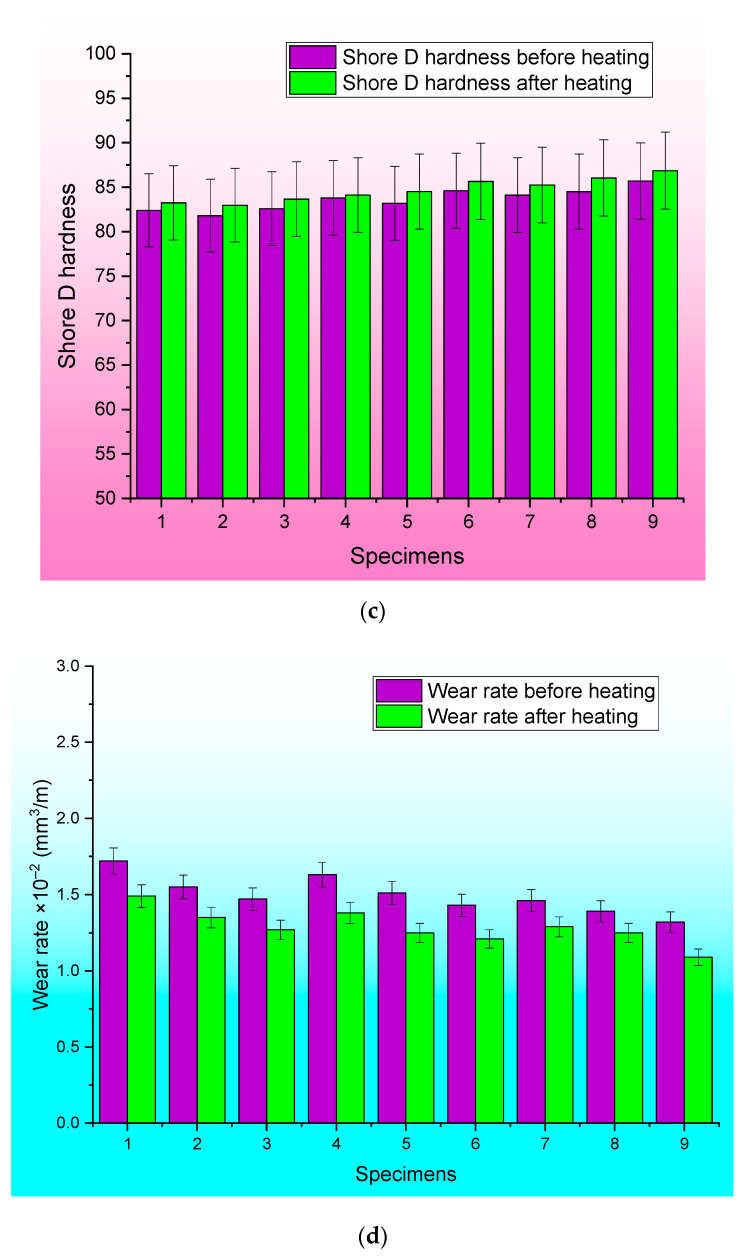
Comparison of responses of original and post-heated specimens: (**a**) tensile test, (**b**) flexural test, (**c**) Shore D hardness test, and (**d**) wear rate.

Figure 7a–d demonstrate the differences in the properties exhibited by the original specimens and the specimens that were subjected to post-heating.

**Figure 8:**
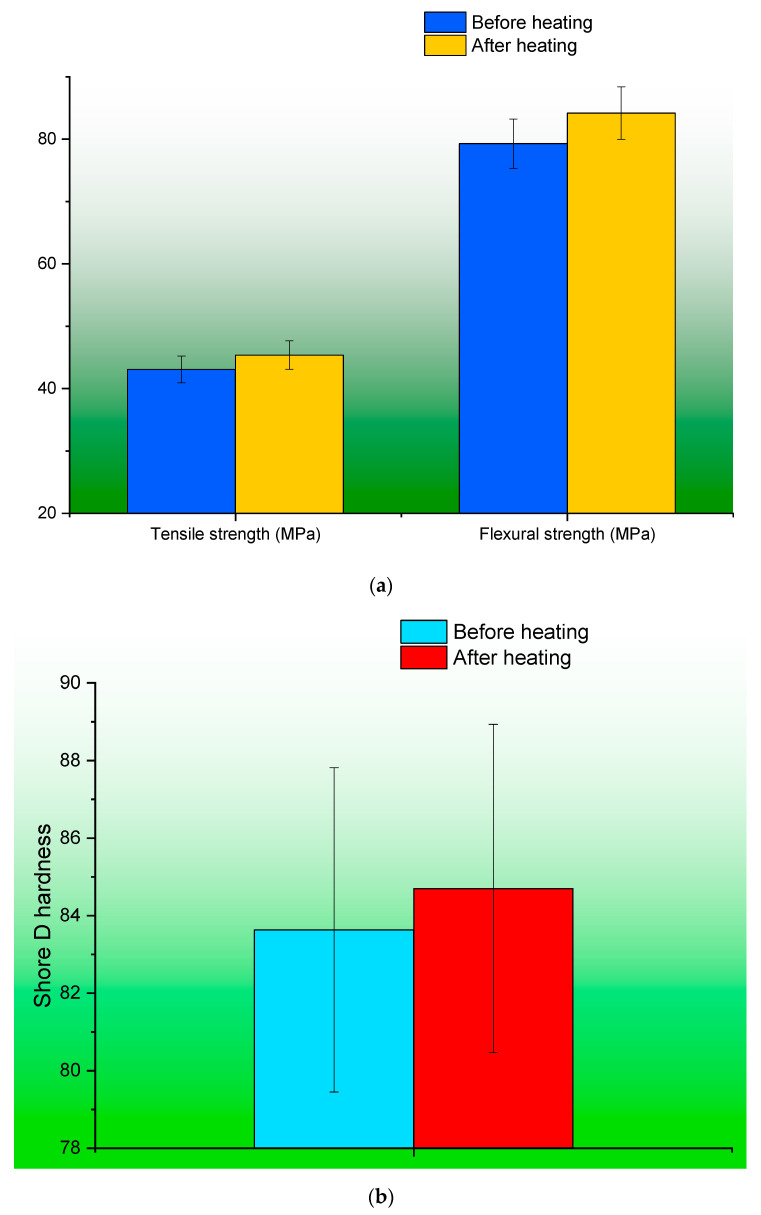

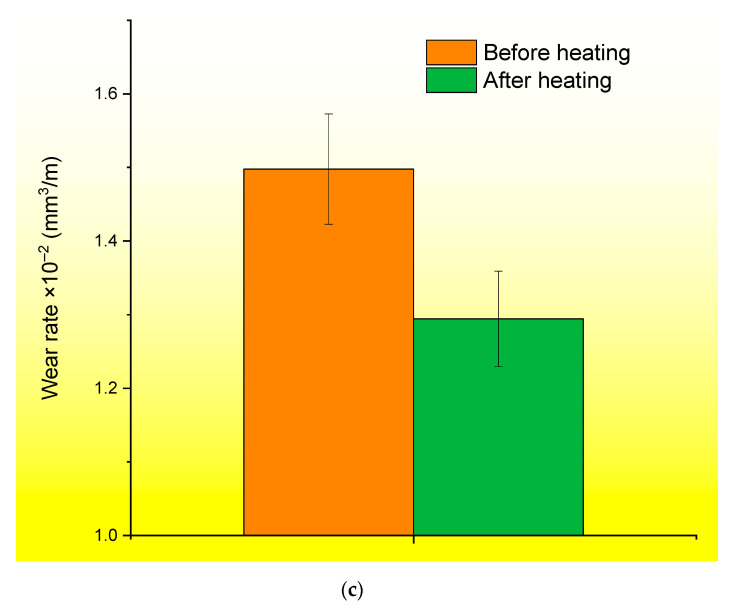
(**a**) Variations in the average values of tensile, flexural properties of specimens before and post heating; (**b**) variation in hardness before and post heating; (**c**) variation in the rate of wear before and post heating.

Figure 8a shows the average difference in the tensile and flexural properties of the original and post-heated specimens. The specimens that underwent post-heating exhibited a slight enhancement in their features, although without a notable level of statistical significance. The addition of filler may lead to the formation of internal agglomerations and cavities between the additive and the matrix. Following the printing procedure, a subsequent heat treatment is implemented to address the presence of micro voids or cavities that may have emerged. The application of heat treatment resulted in the consolidation of the printed layers, hence improving the overall surface quality of the samples. Consequently, the presence of voids that had the potential to induce structural distortion under the influence of an applied load were effectively remedied, thereby enhancing the overall mechanical robustness. Figure 8b,c depict the disparity in the mean values of Shore D hardness and wear rate prior to and after to the application of heat. The marginal increment in hardness exhibited a direct impact on the rate of wear, as evidenced by the corresponding reduction in the wear rate.

**Figure 9:**
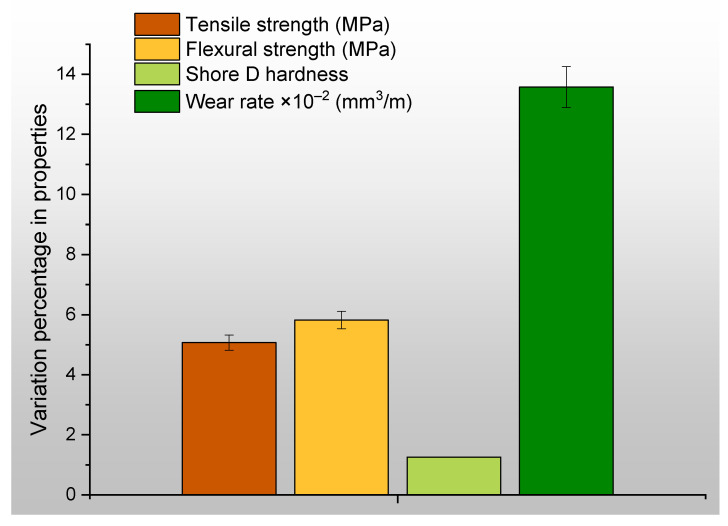
Percentage variation in mechanical properties of PLA-Al specimens before and after heating.

Figure 9 displays the percentages of variance that are associated with specific characteristics. After subjecting the original specimens to the heating procedure, it was observed that the wear rate exhibited a significant decrease of 13.6% in comparison to the initial measurements. This reduction in wear rate was identified as the most substantial change observed. Almost all of the assessed characteristics exhibited a modest increase in level as a result of the post-heating treatment. In the future, not only should the effect of heating be evaluated, but also the effect of cooling should be taken into consideration. During the course of this research, the specimens were subjected to step-by-step increases in temperature as the experiment progressed. Once the specimens had reached the desired temperatures, they were then cooled by being exposed to the ambient air. Because of the amount of cooling time that was granted, the specimen may have varying degrees of both hardness and ductility. In upcoming research, specimens will be categorized, subjected to variable cooling techniques, and analyzed for changes in their properties.

## 4. Regression Analysis

Regression analysis is a statistical tool used to find the most influencing variable among the input variables, and also it shows the contribution of each input factor in determining the output responses. The regression coefficient depicts the significance of the model, and plots like main effect, interaction, and contour indicate the influence, dependency, and optimum input factors for desired outputs, respectively. The ANOVA table represents the Fischer value, which indicates the contribution levels of the each chosen input variable. As the maximum results were obtained after heat treatment, the experimental results obtained after heat treatment were taken for optimization. MINITAB software (MINITAB 19) was used for the regression analysis. Tensile, flexural, and hardness properties are expected to be maximal, and the wear rate was expected to be minimal. The main effect plots were plotted based on this desirability. The Fischer values obtained through the ANOVA table are displayed in Table 4, which indicates the influence of each input variable on the results. From Table 4, it is evidenced that the print angle and the air gap are the two most influencing factors, and the printing pattern has a minimal influence on the responses. The ANOVA and the summary tables for all the tests are included in the supplementary data for the reference. Regression coefficients for tensile, flexural, hardness, and wear rate were recorded as 97.49%, 94.61%, 97.75%, and 98.11%, respectively. As the regression coefficients for all the properties were obtained above 95%, the model was said to be significant. Figure S1a–c, provided in the Supplementary Data, depict the plots for the tensile strength values, from which it can be understood that the larger the S/N ratio the better, and also that the influence of the print angle and the air gap is significant in determining the responses. Based on the analysis of the interaction plot, it can be observed that there exists a significant relationship or dependence among the input variables. A contour plot was generated to illustrate the relationship between the two most influential parameters, which are expected to yield optimal outcomes. The optimal conditions for achieving optimum tensile strength in the printed object involve adhering to a print angle of 45° and utilizing a negative air gap.

There was no much difference between the plots of tensile and flexural results. The main effect and the interaction plot and contour plots remains same for tensile and flexural results. Figures S2 and S3, alongside to the Supplementary Data, shows plots related to the flexural test results and Shore D hardness results. The factor that had the most influence on achieving maximum hardness was determined to be the print angle. The interaction plot revealed that there was a dependency between the air gap and fill pattern, while the other factors were found to be independent. The factor that exerted the most influence on minimizing wear rates was determined to be the air gap, and it was observed that there was a dependency among all the input factors. The air gap must be negative and the print angle must be 45° in order to achieve minimal wear rates. Figure S4 in the Supplementary Data shows plots from wear tests.

The response optimizer data are represented in the Supplementary Data in Figure S5a–d for tensile, flexural strength, hardness, and wear rate, respectively. Per the predicted data for maximum tensile and flexural responses, the input factors must be A3B3C3. For maximum hardness, the input parameters must be A3B3C1. Again, for minimum wear rates, the input factors must be A3B3C3. The regression analysis findings indicate that the specimens were produced with the intention of attaining optimal outcomes. The utilization of regression analysis data has the potential to enhance printing parameters based on the chosen input parameters. The utilization of these data may facilitate the production of a product that achieves optimal outputs, thereby mitigating the occurrence of unforeseen failures and minimizing wastage.

To confirm the accuracy of the predicted data, per the predicted input factors, the specimens were produced and tested experimentally. Table 5 depicts the validation test data and the error percentage between the predicted and the experimental data. The error percentage was minimal, and within an acceptable range. This indicates the experiments were conducted per the standards, and accurately.

## 5. Macroscopic and Microscopic Analysis

**Figure 10:**
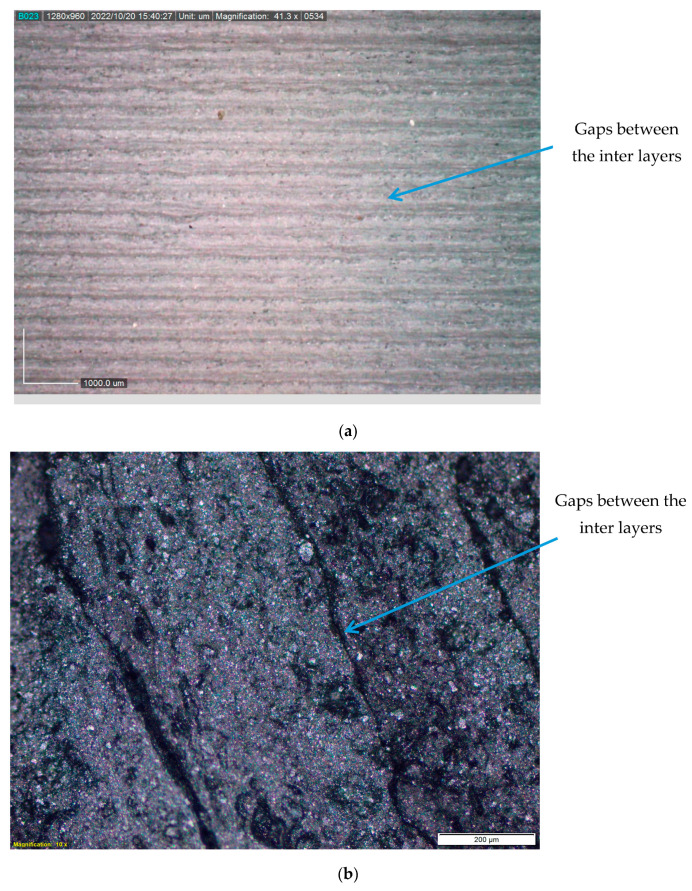

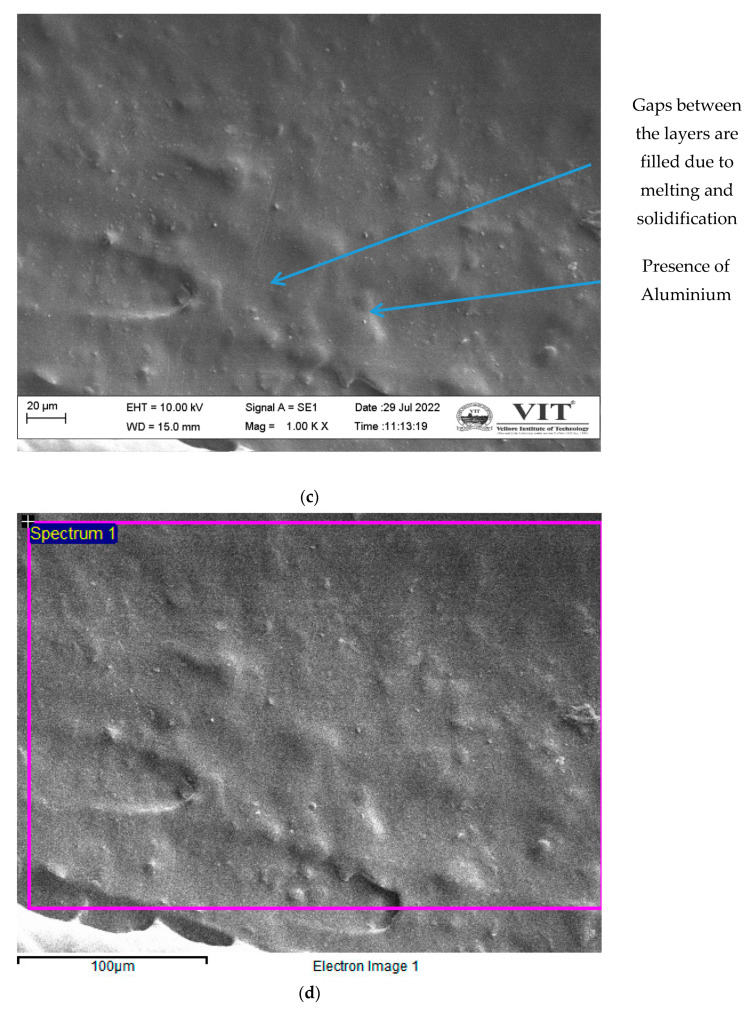

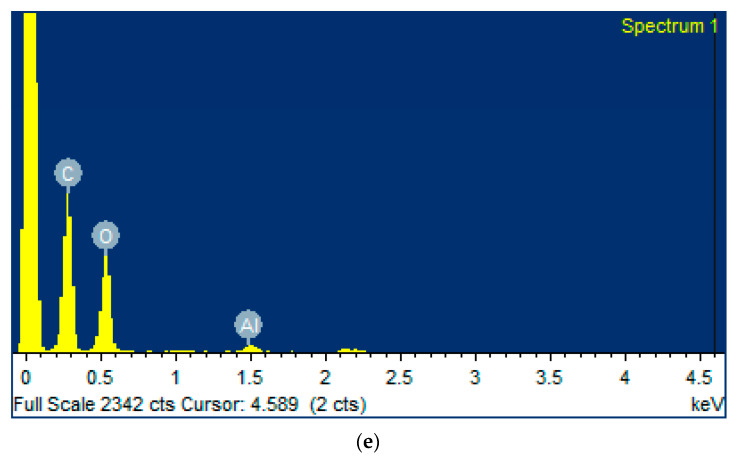
(**a**) Macroscopic image of the PLA-Al specimen; (**b**) microscopic image with higher magnification; (**c**) SEM image of a PLA-Al post-heated specimen; (**d**) electron image of specimen surface from which the EDS data were obtained; (**e**) spectrum of energy-dispersive spectroscopy.

Figure 10a,b show a digital optical microscope image of the specimen surface taken after printing. It is easy to identify the printed layers in this image, and even though the negative air gap is maintained, it is possible to make out the differences between the layers. This will diminish mechanical and surface properties. If the interfacial adhesions between the layers are strong, then the interfacial strength between the layers will also be strong. After being heated, the interface between the layers is successfully fused, as revealed by the scanning electron microscopy (SEM) image in Figure 10c, which shows that it was successfully fused. Over the whole length of the specimen, a smooth surface was discovered, and it was not possible to make out the separation of layers or the gaps that existed between the layers. Additionally, the aluminum fillers that were added to the matrix can be seen; however, agglomeration did not take place, because the filler addition was restricted to a maximum of 2% of the weight. The consequence of post-heating was a smooth surface finish, and any imperfections that had occurred during printing as a result of external variables or non-optimized input parameters were corrected as a result of the process. To confirm the presence of aluminum, energy-dispersive spectroscopy was performed on the sample, and the sample surface is shown in Figure 10d. The spectrum of the energy-dispersive spectroscopy is shown in Figure 10e.

Of the total weight, 1.42% aluminum was found to be present on the area of the particular sample tested, which suggests that the homogeneity of the fillers contained in the matrix was not the same due to specific conditions. Table 6 contains a tabulation of the data obtained from the elemental analysis performed using energy-dispersive spectroscopy.

## 6. Conclusions

In analyzing PLA–Aluminum composite printing process parameters, it was discovered that the specimen with a 45° print angle, a negative air gap, and a honeycomb-type fill pattern had the highest Shore D hardness, tensile, and flexural values, as well as the lowest wear rate. Additionally investigated were the effects of heating the specimens after printing. Still, there was an improvement in mechanical properties after heating the specimens, and it was demonstrated that heating-extruded polymers will improve their mechanical properties to some degree. Post-heated specimens had 46.77 MPa tensile strength, 86.21 MPa flexural strength, 86.86 Shore D hardness, and a minimal wear rate of 1.09 × 10^−2^ mm^3^/m. The tensile strength, flexural strength, and Shore D hardness parameters showed a slight improvement of 5.07%, 5.82%, and 1.26%, respectively. The wear rate of the material exhibited a significant shift, resulting in a reduction of 13.58% in the wear rate. The microscopic images showed a notable enhancement in surface quality, along with the observable integration of printed layers. The performance of PLA with the inclusion of aluminum exhibited more acute results in comparison to pure PLA, potentially due to the incompatibility of the additive with the polymer matrix. In order to ascertain the extent to which the filler enhances electrical and thermal conductivity, it is imperative for forthcoming investigations to quantitatively evaluate these characteristics. Furthermore, it is possible to enhance the additive percentage in order to analyze the impact of additive percentage increments on the properties.

## Figures and Tables

**Table 1:** Process variables and their various stages.

S. No.	Variables	Units	Stages		
			1	2	3
1	Print angle	°	15	30	45
2	Air gap	-	Zero	+ve	−ve
3	Fill pattern	-	Concentric	Honeycomb	Hilbert curve

**Table 2:** Mechanical properties before heating.

Trials	Input Parameters			Output Parameters			
	Print Angle	Air Gap	Fill Pattern	Tensile Strength (MPa)	Flexural Strength (MPa)	Shore D Hardness	Wear Rate × 10^−2^ (mm^3^/m)
1	15	Zero	Concentric	41.5	77.70	82.4	1.72
2	15	Positive	Honeycomb	42.33	78.53	81.8	1.55
3	15	Negative	Hilbert curve	43.44	79.64	82.6	1.47
4	30	Zero	Honeycomb	42.25	78.45	83.8	1.63
5	30	Positive	Hilbert curve	43.48	79.68	83.2	1.51
6	30	Negative	Concentric	43.01	79.21	84.6	1.43
7	45	Zero	Hilbert curve	43.82	80.02	84.1	1.46
8	45	Positive	Concentric	43.43	79.63	84.5	1.39
9	45	Negative	Honeycomb	44.47	80.67	85.7	1.32

**Table 3:** Mechanical properties post heating.

Trials	Input Parameters			Output Parameters			
	Print Angle	Air Gap	Fill Pattern	Tensile Strength (MPa)	Flexural Strength (MPa)	Shore D Hardness	Wear Rate × 10^−2^ (mm^3^/m)
1	15	Zero	Concentric	43.8	81.77	83.24	1.49
2	15	Positive	Honeycomb	44.63	83.03	82.98	1.35
3	15	Negative	Hilbert curve	45.74	85.18	83.67	1.27
4	30	Zero	Honeycomb	44.55	83.11	84.12	1.38
5	30	Positive	Hilbert curve	45.78	83.88	84.51	1.25
6	30	Negative	Concentric	45.31	84.94	85.65	1.21
7	45	Zero	Hilbert curve	46.12	84.67	85.25	1.29
8	45	Positive	Concentric	45.73	84.83	86.04	1.25
9	45	Negative	Honeycomb	46.77	86.21	86.86	1.09

**Table 4:** Fischer values from the ANOVA.

Source	F-Value			
	Tensile Strength	Flexural Strength	Hardness	Wear Rate
Regression	64.65	29.26	72.44	86.68
Print angle	98.82	34.47	178.16	100.17
Air gap	56	48.26	33.28	151.35
Fill pattern	39.12	5.04	5.88	8.52

**Table 5:** Validation test data.

Property	Predicted Levels	Predicted Value	Experimental Value	Error Percentage
Tensile strength in MPa	A3B3C3	47.14	46.82	0.67
Flexural strength in MPa	A3B3C3	86.63	85.92	0.81
Shore D hardness	A3B3C1	86.92	86.87	0.05
Wear rate ×10^−2^ (mm^3^/m)	A3B3C3	1.08	1.06	1.85

**Table 6:** Elemental analysis data from energy-dispersive spectroscopy.

Sl. No.	Elements	Weight Percentage
1	Carbon	50.93
2	Oxygen	47.64
3	Aluminum	1.42

## Data Availability

Data are contained within the article and Supplementary Materials.

## Supplementary Materials

The following supporting information can be downloaded at: https://www.mdpi.com/article/10.3390/polym15244698/s1, Figure S1. Plots for tensile strength. Figure S2. Plots for flexural strength. Figure S3. Plots for Shore D hardness. Figure S4. Plots for wear rate. Figure S5. Optimized responses for each mechanical properties. Table S1. ANOVA table for tensile test. Table S2. Summary table for tensile test. Table S3. ANOVA table for flexural test. Table S4. Summary table for flexural test. Table S5. ANOVA table for Shore D hardness. Table S6. Summary table for Shore D hardness. Table S7. ANOVA table for Wear rate. Table S8. Summary table for Wear rate.
