# Supplementary material for: Optimization of Printing Process Variables and the Effect of Post-Heat Treatments on the Mechanical Properties of Extruded Polylactic Acid–Aluminum Composites
Source: Polymers (Basel). 2023 Dec 13;15(24):4698. doi: 10.3390/polym15244698 (PMC10747175; doi:10.3390/polym15244698)
Supplement: Supplementary file 1 [file polymers-15-04698-s001.zip › polymers-2680074-supplementary.pdf]

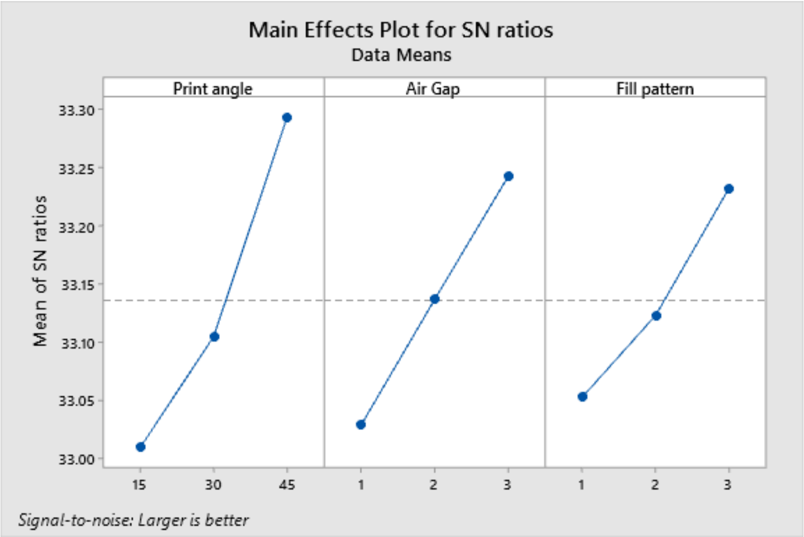
Main Effects Plot for SN ratios
Data Means
Print angle
Air Gap
Fill pattern
Mean of SN ratios
33.30
33.25
33.20
33.15
33.10
33.05
33.00
15
30
45
1
2
3
1
2
3
Signal-to-noise: Larger is better

(a)

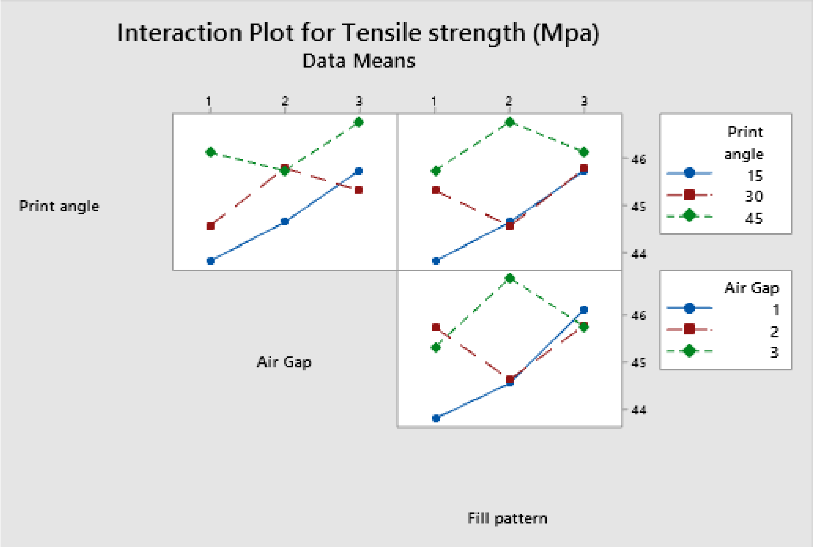
Interaction Plot for Tensile strength (Mpa)
Data Means
Print angle
Air Gap
Fill pattern
Print angle
15
30
45
Air Gap
1
2
3
46
45
44

(b)

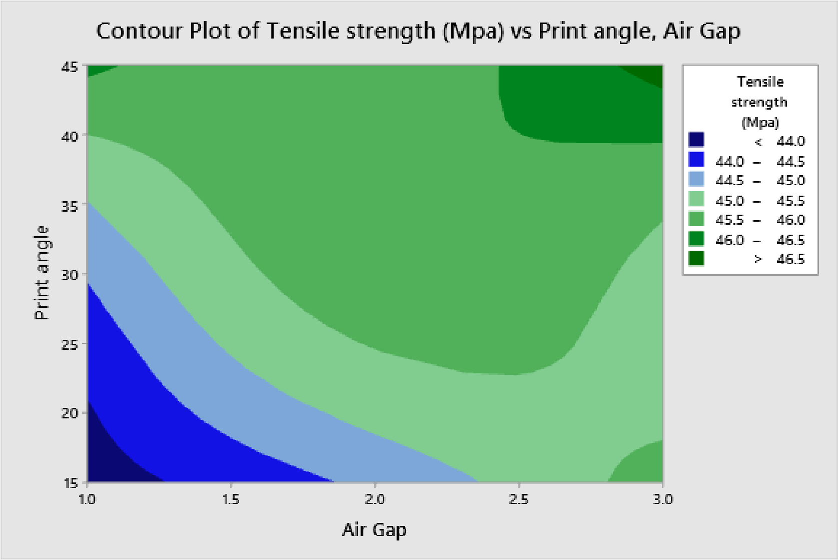
Contour Plot of Tensile strength (Mpa) vs Print angle, Air Gap
Print angle
Air Gap
Tensile strength (Mpa)
< 44.0
44.0 – 44.5
44.5 – 45.0
45.0 – 45.5
45.5 – 46.0
46.0 – 46.5
> 46.5

(c)

**Figure S1.** Plots for tensile strength

**Note:** In the plots, the levels of the Air gap and Fill patterns will be indicated as 1, 2, and 3.
Air gap: 1 – Zero; 2 – Positve; 3 – Negative
Fill patterns: 1 – Concentric; 2 – Honeycomb; 3 – Hilbert curve

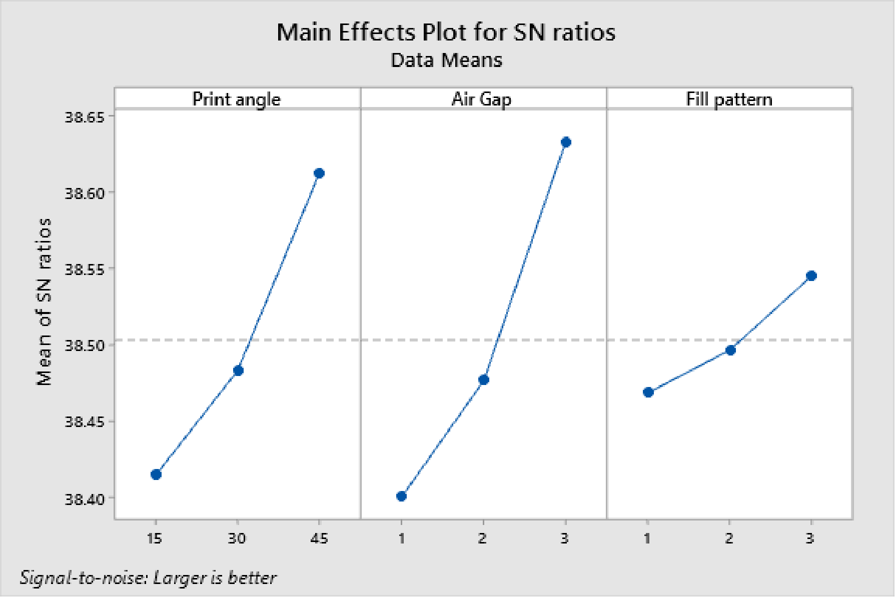


(a)

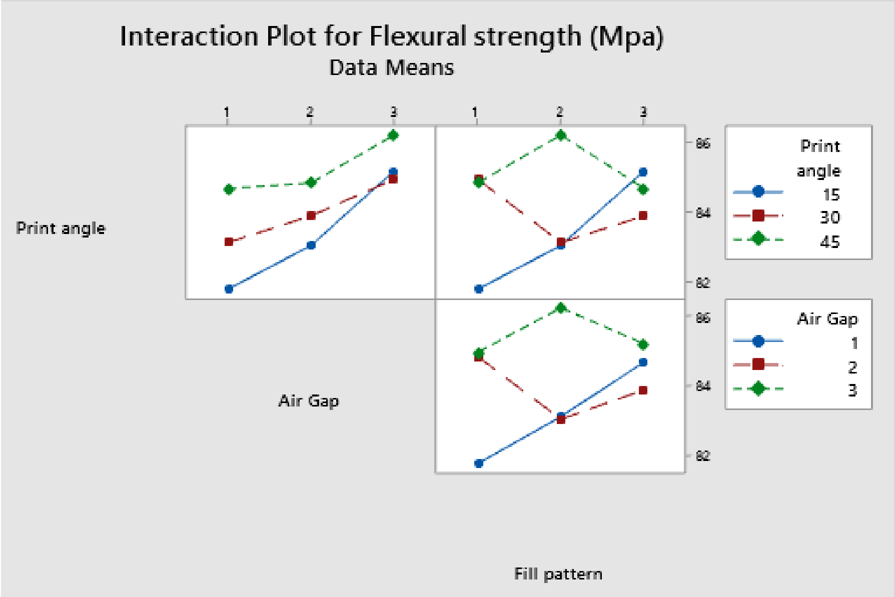


(b)

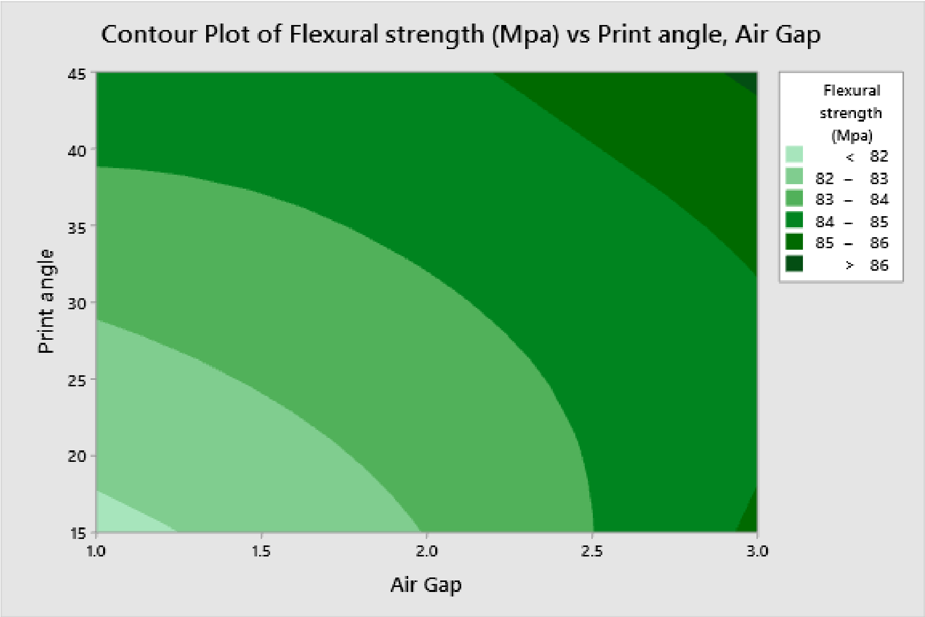


(c)

**Figure S2.** Plots for flexural strength

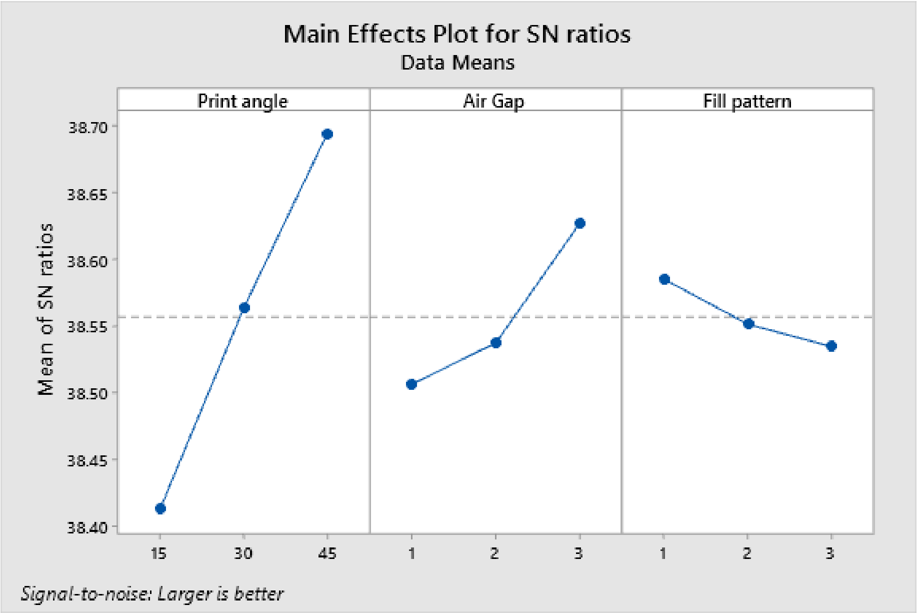
Main Effects Plot for SN ratios
Data Means
Print angle
Air Gap
Fill pattern
Mean of SN ratios
38.70
38.65
38.60
38.55
38.50
38.45
38.40
15
30
45
1
2
3
1
2
3
Signal-to-noise: Larger is better

(a)

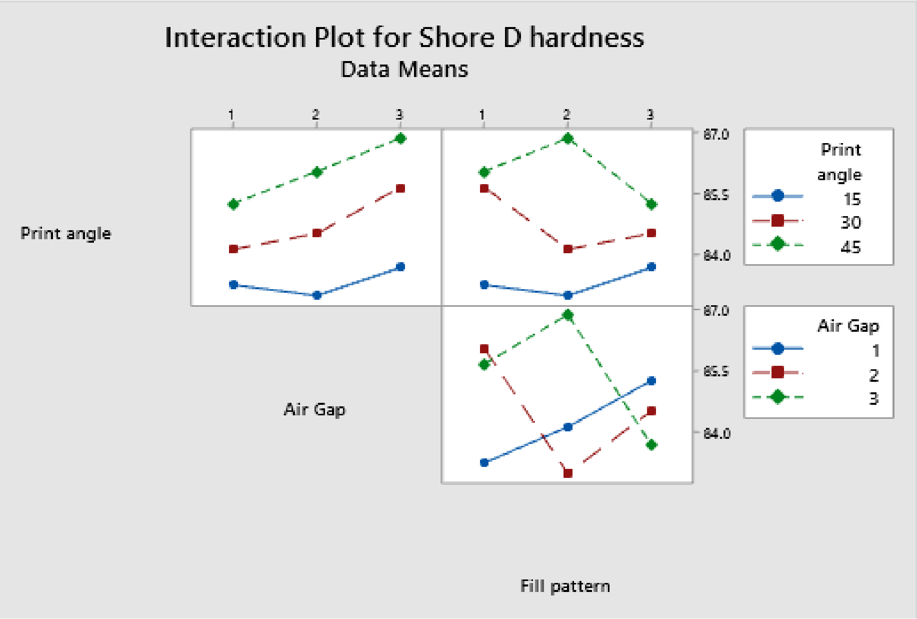
Interaction Plot for Shore D hardness
Data Means
Print angle
Air Gap
Fill pattern
87.0
85.5
84.0
Print angle
15
30
45
Air Gap
1
2
3

(b)

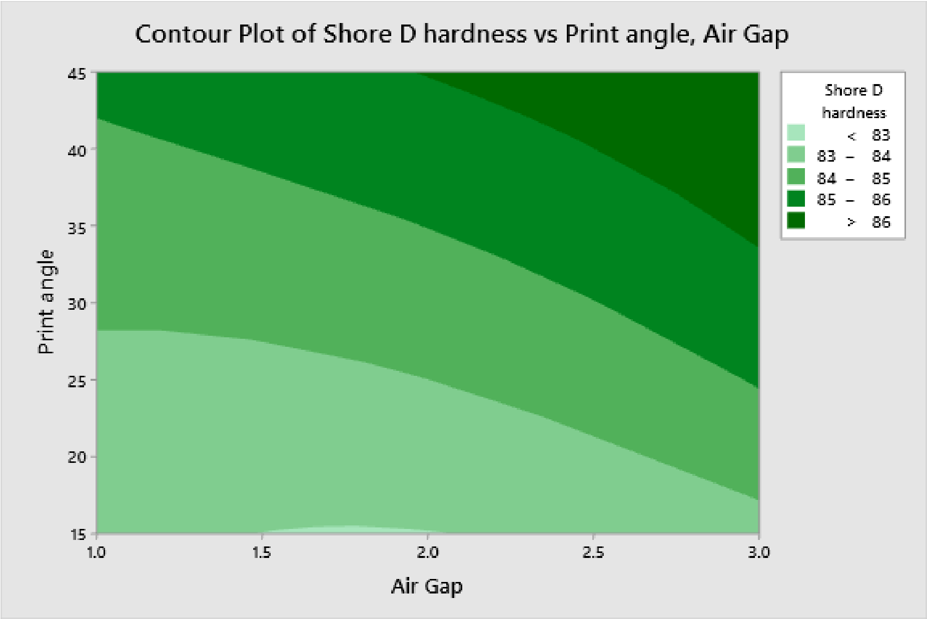


(c)

**Figure S3.** Plots for Shore D hardness

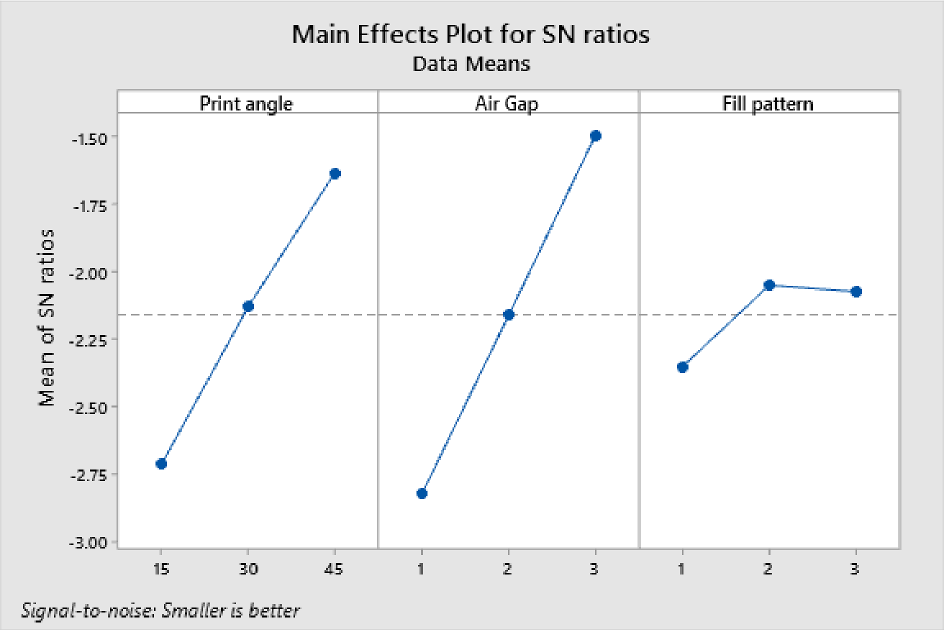
Main Effects Plot for SN ratios
Data Means
Print angle
Air Gap
Fill pattern
Mean of SN ratios
-1.50
-1.75
-2.00
-2.25
-2.50
-2.75
-3.00
15
30
45
1
2
3
1
2
3
Signal-to-noise: Smaller is better

(a)

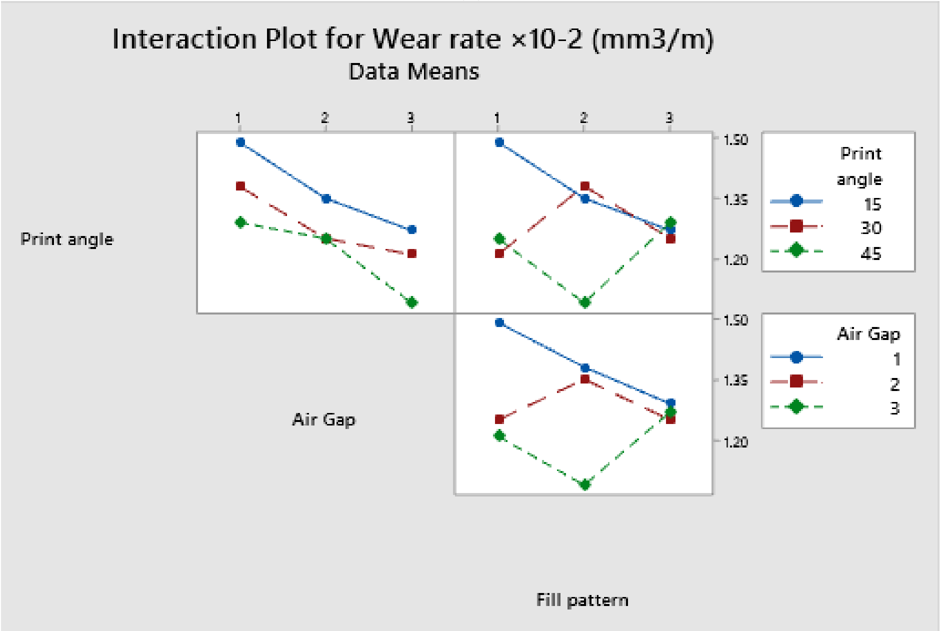
Interaction Plot for Wear rate ×10-2 (mm3/m)
Data Means
1
2
3
1
2
3
1.50
1.35
1.20
Print angle
Print angle
15
30
45
1.50
1.35
1.20
Air Gap
Air Gap
1
2
3
Fill pattern

(b)

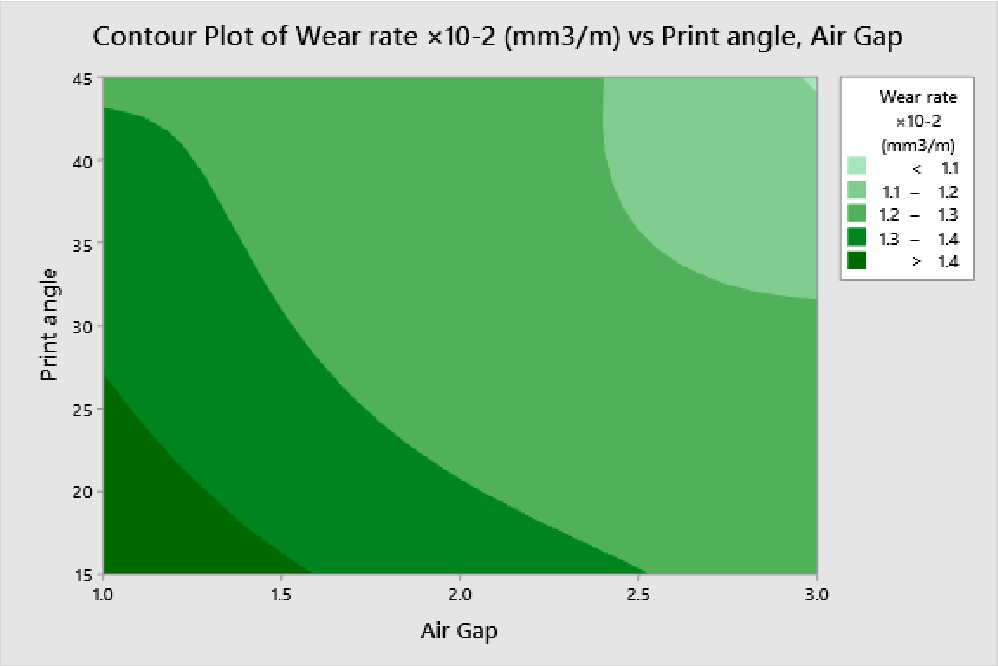

(c)

**Figure S4.** Plots for wear rate

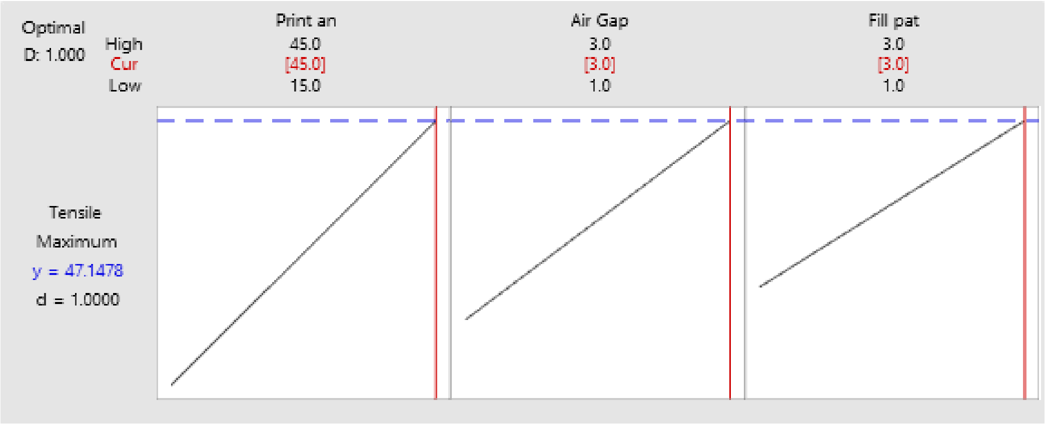

Optimal
D: 1.000
High
Cur
Low
Print an
45.0
[45.0]
15.0
Air Gap
3.0
[3.0]
1.0
Fill pat
3.0
[3.0]
1.0
Tensile
Maximum
y = 47.1478
d = 1.0000


(a)

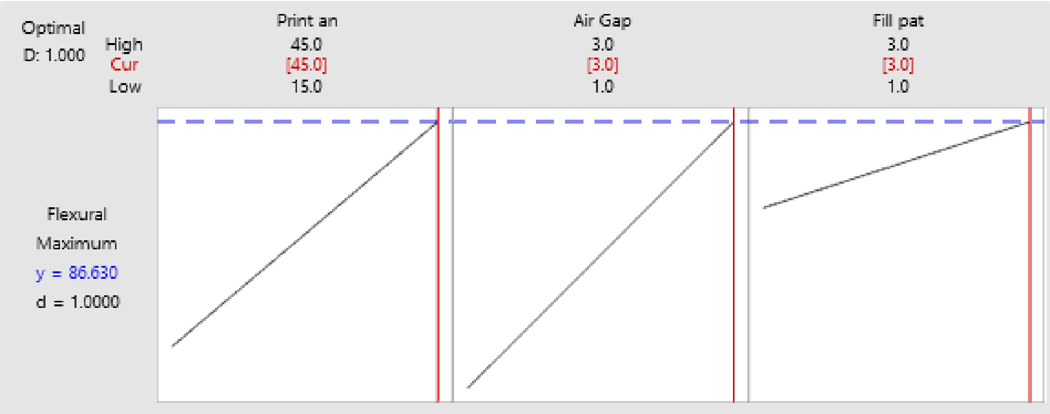

Optimal
D: 1.000
High
Cur
Low
Print an
45.0
[45.0]
15.0
Air Gap
3.0
[3.0]
1.0
Fill pat
3.0
[3.0]
1.0
Flexural
Maximum
y = 86.630
d = 1.0000


(b)

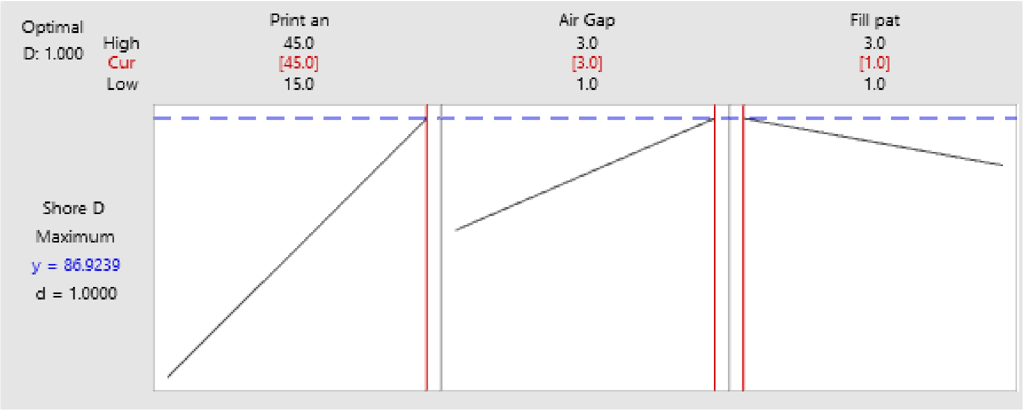

Optimal
D: 1.000
High
Cur
Low
Print an
45.0
[45.0]
15.0
Air Gap
3.0
[3.0]
1.0
Fill pat
3.0
[1.0]
1.0
Shore D
Maximum
y = 86.9239
d = 1.0000


(c)

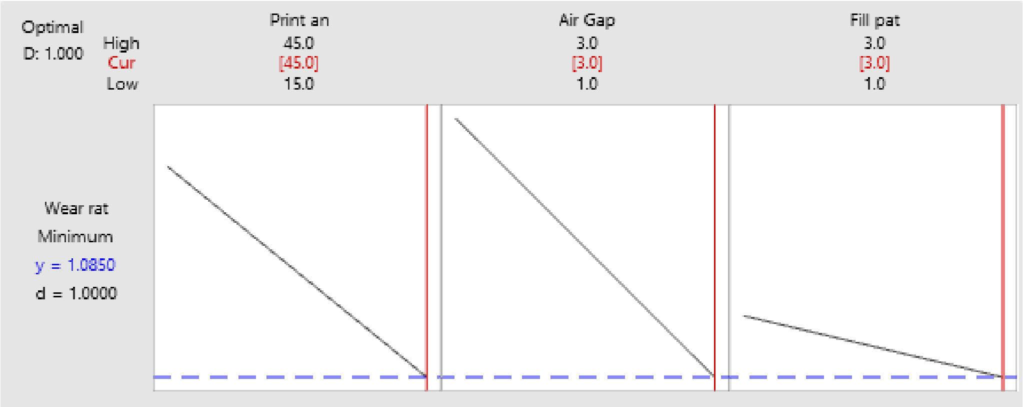


(d)

**Figure S5.** Optimized responses for each mechanical properties

**Table S1.** ANOVA table for tensile test

| Source | DF | Adj SS | Adj MS | F-Value | P-Value |
|---|---|---|---|---|---|
| Regression | 3 | 6.4775 | 2.15917 | 64.65 | 0 |
| Print angle | 1 | 3.3004 | 3.30042 | 98.82 | 0 |
| Air Gap | 1 | 1.8704 | 1.87042 | 56 | 0.001 |
| Fill pattern | 1 | 1.3067 | 1.30667 | 39.12 | 0.002 |
| Error | 5 | 0.167 | 0.0334 | | |
| Total | 8 | 6.6445 | | | |

**Table S2.** Summary table for tensile test

| S | R-sq | R-sq(adj) | R-sq(pred) |
|---|---|---|---|
| 0.182751 | 97.49% | 95.98% | 90.99% |

**Table S3.** ANOVA table for flexural test

| Source | DF | Adj SS | Adj MS | F-Value | P-Value |
|---|---|---|---|---|---|
| Regression | 3 | 13.9329 | 4.6443 | 29.26 | 0.001 |
| Print angle | 1 | 5.4721 | 5.4721 | 34.47 | 0.002 |
| Air Gap | 1 | 7.6614 | 7.6614 | 48.26 | 0.001 |
| Fill pattern | 1 | 0.7994 | 0.7994 | 5.04 | 0.075 |
| Error | 5 | 0.7937 | 0.1587 | | |
| Total | 8 | 14.7266 | | | |

**Table S4.** Summary table for flexural test

| S | R-sq | R-sq(adj) | R-sq(pred) |
|---|---|---|---|
| 0.398422 | 94.61% | 91.38% | 79.94% |

**Table S5.** ANOVA table for Shore D hardness

| Source | DF | Adj SS | Adj MS | F-Value | P-Value |
|---|---|---|---|---|---|
| Regression | 3 | 13.8704 | 4.6235 | 72.44 | 0 |
| Print angle | 1 | 11.3713 | 11.3713 | 178.16 | 0 |
| Air Gap | 1 | 2.1242 | 2.1242 | 33.28 | 0.002 |

| | | | | | |
|---|---|---|---|---|---|
| Fill pattern | 1 | 0.375 | 0.375 | 5.88 | 0.06 |
| Error | 5 | 0.3191 | 0.0638 | | |
| Total | 8 | 14.1896 | | | |

**Table S6.** Summary table for Shore D hardness

| S | R-sq | R-sq(adj) | R-sq(pred) |
|---|---|---|---|
| 0.252642 | 97.75% | 96.40% | 92.25% |

**Table S7.** ANOVA table for Wear rate

| Source | DF | Adj SS | Adj MS | F-Value | P-Value |
|---|---|---|---|---|---|
| Regression | 3 | 0.099683 | 0.033228 | 86.68 | 0 |
| Print angle | 1 | 0.0384 | 0.0384 | 100.17 | 0 |
| Air Gap | 1 | 0.058017 | 0.058017 | 151.35 | 0 |
| Fill pattern | 1 | 0.003267 | 0.003267 | 8.52 | 0.033 |
| Error | 5 | 0.001917 | 0.000383 | | |
| Total | 8 | 0.1016 | | | |

**Table S8.** Summary table for Wear rate

| S | R-sq | R-sq(adj) | R-sq(pred) |
|---|---|---|---|
| 0.019579 | 98.11% | 96.98% | 92.20% |
